# Integrating intensified case finding of tuberculosis into HIV care: an evaluation from rural Swaziland

**DOI:** 10.1186/1472-6963-11-118

**Published:** 2011-05-23

**Authors:** Susan Elden, Timothy Lawes, Søren Kudsk-Iversen, Joris Vandelanotte, Sabelo Nkawanyana, William Welfare, John Walley, John Wright

**Affiliations:** 1 Nuffield Centre for International Health and Development, Institute of Health Sciences, University of Leeds, Leeds, UK; 2 Good Shepherd Hospital, Siteki, Swaziland; 3 Department of Health Sciences, University of York, York, UK; 4 University of Sheffield School of Medicine and Biomedical Sciences, Sheffield, UK; 5 International Center for AIDS Care and Treatment Programs, Mbabane, Swaziland; 6 Bradford Institute for Health Research, Bradford Teaching Hospitals, UK

## Abstract

**Background:**

Swaziland has the highest HIV prevalence in the world and the highest estimated tuberculosis incidence rate in the world. An estimated 80% of TB patients are also infected with HIV. TB detection through intensified case finding (ICF) has yet to become a routine aspect of integrated tuberculosis and HIV care. The purpose of this study was to evaluate implementation of ICF for TB into routine integrated tuberculosis and HIV care at 16 community clinics and one district hospital in Swaziland.

**Methods:**

Nurses and lay counsellors conducted ICF using a TB screening tool and patient pathway at all HIV service entry points in clinics and the hospital. The patient pathway had three-stages; screening, sputum smear diagnosis and TB treatment initiation. Outcomes and losses to follow up were monitored at each stage. Patient demographics, access, and service feasibility and effectiveness were compared at hospital and clinic sites.

**Results:**

1467 HIV patients at clinics and the hospital were screened over a 3 month period. Large losses to follow up occurred prior to the sputum diagnosis stage; only 47% (n = 172) of TB suspects provided a specimen. 28 cases of smear positive TB were diagnosed and 24 commenced treatment. People screened at clinics were significantly more likely to be female, older, and from rural or geographically remote areas (p < 0.001). There was no significant difference between the hospital and clinics sites in the proportion of all participants screened who were smear positive (x2 = 1.909; *p = 0.16*). The number needed to screen to detect one sputum positive TB case was 34 at clinics and 63 at the district hospital.

**Conclusions:**

ICF was operationally feasible and became established as a routine aspect of tuberculosis and HIV integrated care. ICF in community clinics was potentially more accessible to an underserved, rural population and was as effective as the hospital service in detecting smear positive TB.

## Background

Tuberculosis remains the leading cause of morbidity and mortality amongst people living with HIV/AIDS worldwide [1]. Of the 1.37 million people with active TB who are also co-infected with HIV worldwide, 80% live in Sub-Saharan Africa [2]. In 2007 the tuberculosis case detection rate in the Africa Region was 47%. This remains far short of the Global Stop TB Strategy target of 70% of incident smear-positive cases detected and treated in DOTS programmes [3]. Passive case finding through directly observed short-course strategy (DOTS) is no longer seen as an adequate response to the rising TB incidence rates within generalised HIV epidemics [4,5].

A response to this global challenge was outlined by the World Health Organisation (WHO) in its "Three Is" policy: Intensified case finding (ICF), isoniazid prevention therapy (IPT), and infection control [6]. ICF aims to provide early detection thus increasing the chance of survival in TB infected individuals. ICF is also an important public health intervention, by aiming to reduce disease within health services and community settings, transmission to the wider population should also be reduced [5-7]. Despite this, scaled up delivery has progressed slowly with estimates from 2008 showing only 4.1% of HIV positive people screened for TB [8]. Lack of data to inform policies, poor system infrastructure and concerns about feasibility and human resources have constrained wider implementation in high HIV prevalence settings [5,9].

Swaziland has the highest HIV prevalence in the world with 26% of the adult population infected [9] and the highest estimated TB incidence rate in the world at 1,198 per 100,000[10]. An estimated 80% of TB patients are also infected with HIV [10]. The high HIV prevalence and TB/HIV co-infection rates pose serious challenges to health care delivery systems. Despite national guidance encouraging ICF in all HIV testing and counselling settings, detection has yet to become a routine part of TB/HIV integrated care [10]. This is especially true at the primary care level where most people first make their debut into the healthcare system through HIV testing. Additional constraints include human resources and a limited number of TB laboratory diagnostic facilities [11,12].

This study implemented and evaluated a programme of ICF into a high HIV prevalence, low resource, rural setting. The objectives were to determine whether ICF was operationally feasible in the hospital and clinic settings, to measure and compare outcomes at both sites, examine the barriers to access, and assess the potential for scale up of ICF.

## Methods

### Setting

Implementation took place in the Lubombo region; the poorest and most rural region in Swaziland with population of 208,000. The ICF programme was implemented at the one district hospital which serves the region and the 16 community clinics which provided HIV services. The other clinics within the region which did not provide routine HIV testing or follow up care were not involved.

Prior to this study, community clinics did not provide TB screening. TB diagnosis and treatment was provided at the district hospital's TB unit, with some community clinics involved in TB treatment follow up. TB care was not integrated within HIV services. In the hospital TB care had been partially integrated within HIV care when patients were screened for TB as part of their commencement of ART. However, in both the district hospital and the community clinics, TB screening was not routinely or systematically provided to patients who were receiving HIV testing or follow up pre ART care.

The district hospital TB diagnostic services included smear microscopy and X-ray. Prior to the study, HIV services at the district hospital included provider initiated HIV testing and ART treatment. ART commencement was provided by a physician on the basis of WHO clinical staging, CD4 results and patient adherence to treatment.

HIV and TB services were provided separately and TB screening and investigations were not routinely provided in HIV testing and pre-ART care. Patients commencing and receiving ART had some screening through the physician but this was not formally recorded or monitored.

In community clinics, prior to the study, partial HIV services were provided. This included provider initiated HIV testing and CD4 counts, via weekly transportation system from the district hospital to the clinics. Patients commenced on ART at the district hospital were eligible for "ART Roll out" care at the community clinics. This consisted of routine follow-up care and medication refills at the clinics through the service. Routine TB screening or diagnosis was not provided at community clinics. Instead, patients were referred to the district hospital for microscopy and X-ray.

### ICF programme design

**Figure 1:**
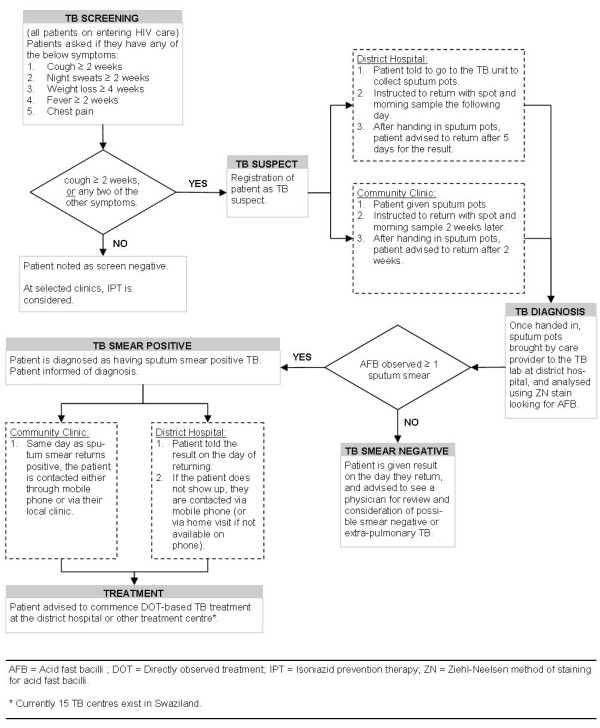
Intensified case finding programme and pathway

The ICF programme included four elements: ICF pathway and screening tool (Figure 1); staff training; transportation system for sputum specimens and smear results; and a reporting and monitoring system.

A three-stage patient pathway (Figure 1) was designed which included: 1) TB screening all HIV patients attending care; 2) laboratory diagnosis and patient notification; and 3) TB treatment initiation for all sputum-smear positive cases. Nurses and lay counsellors used a five-question screening tool validated by the Swaziland Ministry of Health to assess TB symptoms at each HIV diagnosis, pre-ART care and treatment visit. The screening tool had already been introduced into some ART services across the country as part of TB/HIV integrated care. People with symptoms of TB were requested to submit two sputum specimens (one spot and one morning sputum) for smear microscopy for acid-fast bacilli (AFB). Those at the district hospital were requested to return the following day with sputum specimens. At clinic sites patients were asked to return specimens by the date of the bi-monthly specimen collection. Patients screened at the hospital returned after five days to collect smear results. Clinic patients with sputum-smear positive results were informed by telephone, via the community clinic or home visit by outreach worker, ideally within five days. Negative results were returned at the next bi-monthly transportation. All sputum-smear positive cases were instructed to come to the district hospital in order to commence TB treatment.

Staff training consisted of a standardised two day training course for nurses and HIV counsellors at clinics or the district hospital. Training was based upon the National TB screening tool and ICF pathway. After training, we then made monthly visits to each implementation site to ensure correct and consistent usage of ICF methods.

A bi-monthly transportation service was set up to transport sputum specimens from clinics to the district hospital laboratory and to return results.

**Figure 2:**
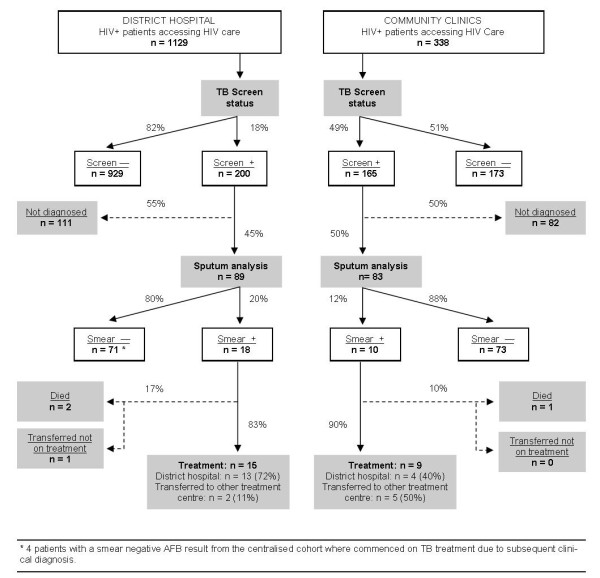
Summary of intensified case finding outcomes by screening cohort

A standardised reporting and monitoring system recorded patient demographics and clinical information at first visit and patient outcomes at screening, diagnosis and treatment initiation (Figure 2). This permitted ongoing assessment of programme performance and losses to follow up amongst the screening cohorts.

One full-time registered nurse and one part-time TB/HIV Outreach Coordinator were hired to coordinate the regional ICF programme and provide transportation. The equipment required for the programme included sputum pots, TB screening tools and patient registers provided by the National TB Programme. The registered nurse, outreach coordinator and hospital TB department coordinating the programme all received cellular phones with monthly credit. Clinics were given monthly cellular telephone credit to contact the hospital.

### Study population and data collection

The ICF programme started in February 2009. We followed a cohort of HIV positive patients screened as part of routine HIV testing or pre-ART services in a three month period (March to May 2009) at the district hospital (n = 1129) or community clinics (n = 338). The patient follow-up period ended at the end of July 2009. All HIV positive outpatients attending HIV testing or pre-ART services at the district hospital or community clinics were included in the analysis.

Patients on antiretroviral treatment were excluded as TB screening had previously been established within the ART service. Hospital inpatients were not included. Other exclusion criteria included those already diagnosed with TB, currently on TB treatment, or having taken treatment for TB in within 3 months before screening (n = 19).

### Analysis

We calculated the proportion of smear positives and the number needed to screen (NNS) to achieve one smear-positive diagnosis or treatment initiation to determine the ICF yield. We analysed the probability of initiating TB treatment within one month of diagnosis using logistic regression. Proportions of patients starting TB treatment after diagnosis was analysed using Yates corrected chi-square tests. Distance, time and cost of travel were estimated using a two-week sample of clinic attendees (n = 85). Data on patient demographics, residence, and outcomes of TB screening were derived from routinely collected information on TB registers. Travel costs were derived from local bus companies and healthcare workers and converted from local currency (Emalangeni) to international dollars ($pop) using World Bank conversion rates.

This study was reviewed by the ethics committee at the University of Leeds and approved by the Swaziland Ministry of Health and Social Welfare. It was an evaluation and audit of routine service development rather than a research study and individual patient consent was not required

## Results

### Performance through the pathway

During the 3 month period, 1467 HIV patients were screened for TB in the ICF programme. Of those screened, 365 (25%) were identified as TB suspects. The major loss in follow up occurred prior to laboratory diagnosis (Figure 2). Of those with symptoms of TB, only 172 (47%) provided sputum samples for smear analysis. Twenty eight individuals (16%) submitted sputum-smear positive samples. Amongst those who were smear positive, 24 (86%) commenced treatment, 3 died prior to commencing treatment, and 1 delayed treatment beyond one month of diagnosis (Figure 2). Using the total number eligible for screening (n = 1467) as a denominator, the proportion diagnosed with smear positive TB was 2% (28/1467) overall giving a number needed to screen (NNS) of 52.

### Comparison of hospital and clinic sites

The proportion of individuals identified as TB suspects was significantly higher in community clinics (c.49%) than at the district hospital (c.18%); chi squared 133; *p < 0.001*. There was no significant difference between the hospital (20%) and clinic sites (12%) of those who were smear positive amongst those who submitted sputum. (X2 = 1.55; *p = 0.21*).

The proportion of people starting treatment within one month of a smear positive diagnosis was not significantly different between the hospital and clinic groups (90% and 83%) (x2 = 0.024; df = 1; *p = 0.8769*)(Figure 2).

The number needed to screen (NNS) in order to detect one new case of AFB smear positive TB was 34 cases in clinics as compared with 63 cases in the hospital. The NNS for one treatment initiation was 38 in clinics and 75 in hospital.

### Demographics and characteristics of individuals diagnosed with TB

Those screened at clinics were significantly more likely to be female, older, and live more geographically remote to the district hospital (*p *< 0.001) (Table 1). The age and sex of participants approximated the regional and national HIV positive and TB populations. Older age (OR (95%CI) for 10 years = 1.11 (1.02-1.20); *p  = 0.018*) and male gender (OR (95% CI) = 1.78 (1.36 - 2.34); *p < 0.001*) were predictors of a smear positive result in both hospital and clinic settings.

**Table 1:** Characteristics by screening location and comparison by univariate logistic regression

Patient Characteristic	District hospital n = 1129	Community Clinic n = 338	Univariate analysis
	**No (%)/Mean (S.D)**	**No (%)/Mean (S.D)**	**P-value‡**

Gender			0.000

Male	411 (36.4%)	80 (23.7%)	

Female	717 (63.6%)	258 (76.3%)	

Age (yrs)	35.2 (14.6)	38.8 (13.8)	0.000

Region			0.000

Lubombo	997 (88.3%)	313 (92.6%)	

Other region	122 (10.8%)	1 (0.3%)	

Unknown*	10 (0.9%)	24 (7.1%)	

Distance to screening centre (km)	39.9 (28.8)	18.55 (13.50)	0.000

Cost of travel to screening centre ($Int***)	5.0 (3.8)	3.6 (0.0)	0.000

Remoteness to district hospital (km)	39.9 (28.8)	56.24 (18.33)	0.001

Cost of travel to district hospital ($Int***)	5.0 (3.8)	8.3 (3.1)	0.000

‡ Wald test

* Patients with no data on region or residence did not differ significantly from the remainder of the sample across any other variable.

*** International dollars adjusted for purchasing power parity

## Discussion

This evaluation study found that ICF can be feasibly implemented into routine HIV services. Provision of ICF in both clinic and hospital settings was effective in detecting smear positive TB. The proportion of observed smear positives amongst those in the study was 2% (NNS 52). This is lower than findings from other studies. In a systematic review, ICF in ART and medical clinics in sub Saharan Africa had a median detection rate of 8.6% (range 3.6-24.7) with a NNS of 12 (4-28) [5]. The lower proportion of observed smear positives may be explained by a reliance on sputum smear diagnosis alone (without the use of chest x-ray), or that screening criteria was not sufficiently sensitive to detect cases. It is important to note that our observed analysis excluded over half (53%) of TB suspects who did not return sputum specimens. If the assumption was made that those who did not submit sputum specimens were just as likely to have smear positive TB as those who did submit, then the yield of smear positives would be 4% (61/1467) which is similar to rates found in other published studies [5]. However, this would not have reflected the reality of implementing an ICF programme in a low resource, rural setting and the practical messages about motivating patients to provide specimens.

The proportion of individuals screened being identified as TB suspects was significantly higher in the clinics (49%) than in the hospital (18%) *p < 0.001*. One explanation could be that staff were more motivated or there was more rigorous application of the screening tool at some clinics.

The key barrier in the pathway was that 53% of TB suspects did not return sputum specimens. This drop out was in the ICF programme and not overall HIV follow up care. The potential reasons include: financial and geographical barriers for patients in returning sputum to clinics or hospital, difficulty in producing sputum, or patients not prioritising this. One potential solution would be increased use of spot sputums, sputum induction and, at the hospital, real time microscopy to reduce the loss between submission and diagnosis.

In this ICF programme, TB treatment was available only at major centres including the district hospital. To maximise the benefits of ICF in community clinics, it may be necessary to decentralise TB treatment as well as screening.

Patient demographics showed that providing ICF at both clinics and the hospital may improve access to a wider section of people living with HIV. Clinic-based ICF improves TB detection rates in specific populations, namely rural and older. Therefore, providing ICF at both sites may provide additional benefit in early detection and reduced transmission among populations which are inter-connected.

### Strengths of evaluation

This was a pragmatic evaluation in a low resource setting which provides timely evidence about an important new public health programme. Evidence based research must draw on a range of designs and methodologies in order to inform policy and practice. We sought to balance research rigour with practical implementation and evaluation. We chose to simplify the intervention, minimise data collection and not to place additional workload demands onto an already overstretched and understaffed primary health care service. Our goal was to incorporate ICF as an integral part of TB/HIV care within the existing services and our results are likely to be generalisable to real world rural African settings.

### Limitations of evaluation

Key issues included the study design and methodology, the small sample size of those initiating TB treatment and our ability to follow those patients beyond treatment initiation. These factors limited our ability to interpret findings with certainty and to provide wider generalisations. Extending the time period, following the patients through to treatment completion, and additional qualitative work would have provided additional evidence and a greater understanding of the facilitators and barriers to implementation.

We were unable to record the denominator or total number of people eligible during this time period, due to the reliance on routine data and the limitations of data quality. It may be that these patients would present clinically over time irrespective of ICF but our aim was to detect these earlier before clinical deterioration and further onwards transmission. We were also unable to distinguish or disaggregate newly diagnosed HIV positive patients from those with a known diagnosis who had been in care. Because of this, it is difficult to provide a stronger explanation for the low proportion detected amongst those screened and the higher proportion (16%) of smear positives amongst TB suspects.

Another limitation was our analysis of smear positive TB outcomes only. We followed the 28 smear positive patients through to treatment initiation, rather than following up the outcomes all 365 TB suspects, or in fact, all 1467 people screened. A recent systematic review found that, amongst HIV co-infected patients, a significant proportion of TB cases may remain asymptomatic or present with minor symptoms [5]. Alternative approaches include requesting sputums from all new HIV diagnoses regardless of symptoms or to perform additional investigations such as sputum culture or chest radiography on those with positive screening. This measure may have increased the yield of TB cases detected; however, it was balanced against the additional costs.

Several challenges to sustainability and scale up were identified by this study. Transportation, laboratory facilities and reporting systems remain key challenges for service expansion. ICF is reliant on having an effective and efficient TB programme for patients to enter and ensuring that patients complete treatment. Expanding and scaling up ICF nationally may pose an additional strain on existing services. The Swaziland National TB Control Programme is currently faced with human and financial resource constraints, insufficient TB/HIV coordination, and limited laboratory capacity. ICF must be strengthened alongside our efforts to improve the necessary capacity, infrastructure and efficiently of the National TB Control Programme.

## Conclusions

This study found that ICF can be feasibly implemented into routine TB/HIV services in clinics and hospitals in a rural high HIV prevalence setting. ICF was as effective in clinics as in the district hospital and was potentially more accessible to an underserved rural population. The lessons from this study may inform policy and practice in Swaziland and also inform the evidence base on the facilitators and challenges to integrated TB/HIV care in other high prevalence HIV settings across Southern Africa.

## Competing interests

The authors declare that they have no competing interests.

## Authors' contributions

All authors contributed to the evaluation of the programme. TL carried out the statistical analysis. All authors read and approved the final manuscript.

## Pre-publication history

The pre-publication history for this paper can be accessed here:

http://www.biomedcentral.com/1472-6963/11/118/prepub
